# Factors associated with psychosocial problems in Korean nursing and non-nursing students during the COVID-19 pandemic

**DOI:** 10.7717/peerj.12541

**Published:** 2021-12-07

**Authors:** Jeongmin Ha, Dahye Park

**Affiliations:** 1 Department of Nursing, Chung-Ang University, Seoul, South Korea; 2 Department of Nursing, Semyung University, Jecheon-si, Chungbuk, South Korea

**Keywords:** Psychosocial problems, Korean, Nursing students, Non-nursing students, COVID-19 pandemic

## Abstract

**Background:**

The coronavirus disease 2019 (COVID-19) pandemic has changed our lives in many ways, including school closures and social distancing practices. These abrupt life changes may have led to psychosocial problems in college students. This study aimed to identify the factors associated with psychosocial problems in South Korean nursing and non-nursing students.

**Methods:**

This descriptive cross-sectional survey was conducted with 139 nursing and 147 non-nursing students (*N* = 286) between August 6 and October 30, 2020. We investigated participants’ general characteristics (that is, sociodemographic and health-promoting behaviors), sensitivity to COVID-19 infection, COVID-19 pandemic response indicators, and psychosocial problems. The factors associated with psychosocial problems were determined using multiple regression analysis.

**Results:**

Among the COVID-19 pandemic response indicators, perceived health status during the COVID-19 pandemic was verified as a factor associated with psychosocial problems in nursing (β =  − 5.831, *p* < .001) and non-nursing students (β =  − 8.513, *p* < .001). Perceived stress (β = 1.263, *p* = .045), trust in policy (β = .892, *p* < .001), and religion (β =  − 1.424, *p* = .004) were verified as correlates of psychosocial problems in non-nursing students.

**Conclusion:**

As the COVID-19 pandemic continues, the stakeholders can use our study results to identify students experiencing psychosocial problems and subsequently as a theoretical background for developing intervention programs for those at a high risk of psychosocial problems. Additionally, it can be used as primary data for future research and practice regarding COVID-19 guidelines among students.

## Introduction

The coronavirus disease 2019 (COVID-19) pandemic resulted in the closure of several educational institutions worldwide (Bourion-Bédès et al., 2021). South Korea (hereafter “Korea”) was no exception, with the government closing schools nationwide owing to the unabated spread of COVID-19. Amid this global public health crisis, students appeared uncertain about the nuances of online education, especially regarding assessment procedures, project implementation, and examinations (Sahu, 2020).

Although observed across all age groups, this uncertainty is particularly relevant to college students since they are at a critical developmental stage—the transition from adolescence to adulthood. This stage is characterized by dynamic growth and drastic changes in social relationships. However, the isolation measures, implemented to contain the pandemic, have restricted the seeking and formation of social relationships. Specifically, college students face travel restrictions, social distancing, and limited or no access to restaurants, cinemas, gyms, museums, and other venues propitious to social gatherings. Such abrupt and drastic changes can lead to psychosocial problems such as depression, anxiety, and substance abuse (Al-Rabiaah et al., 2020). Furthermore, spending prolonged time at home may disrupt the circadian rhythm, leading to irregular sleep patterns, which can intensify psychosocial problems (Naser et al., 2020).

The psychosocial problems among college students have been examined during the COVID-19 pandemic (Xie et al., 2020; Bourion-Bédès et al., 2021; Fu et al., 2021), and differences were found based on students’ majors. They found remarkable differences between medical and non-medical students. For example, a Chinese study found that non-medical students, compared with medical students, experienced more serious psychosocial problems (Xie et al., 2020). In contrast, another Chinese study reported that medical students experienced more severe psychosocial problems, compared with university students (Ye et al., 2020). These contradictory results confirm differences between them in the context of psychosocial problems.

Previous studies have focused only on medical students, examining the relationship between their medical knowledge and clinical practices, and their social and psychological health. Nursing students, like medical students, acquire medical knowledge and practical experience through the nursing curriculum (Günay & Kinç, 2018). Studies performed before the COVID-19 pandemic indicate that nursing students experienced a higher level of stress and anxiety, compared with non-nursing students (Stewart, Greene & Coke, 2018; Zeng et al., 2019).

Despite this, it is difficult to find studies that have explored the differences between nursing and non-nursing undergraduates during the COVID-19 pandemic. In a study that investigated the psychological problems of nursing students after the COVID-19 pandemic began, most participants reported difficulty in concentrating (90%) or anxiety and feeling overwhelmed (84%; Fitzgerald & Konrad, 2021). This may be due to various reasons, including the desire to help others, anxiety regarding their health, maintaining the quality of their education, and completing their education (Swift et al., 2020). Suitable support from professors can be an essential factor in alleviating students’ psychological problems (Fitzgerald & Konrad, 2021). Thus, it is necessary to develop a curriculum that includes customized teaching strategies for both nursing and non-nursing students, through a comparative analysis of their psychological problems.

### Study’s significance

Most experts have projected that the COVID-19 pandemic will likely persist for a long time (Capone et al., 2020), significantly affecting schools and students alike. Accordingly, identifying the risk factors for psychosocial problems in college students during this pandemic is of utmost importance. Since Korean college students have been experiencing severe psychosocial problems (Cho & Lee, 2019; Lee et al., 2021), suicide has become a leading cause of death among young Koreans in their 20 s and 30 s (StatisticsKorea, 2019). However, there is a lack of research regarding factors associated with the psychosocial problems among Korean youth/college students. To bridge this gap, we investigated the correlates of psychosocial problems among Korean nursing and non-nursing university students in the context of the COVID-19 pandemic. The results can serve as a theoretical basis for developing psychosocial intervention programs, helping Korean university students tackle these problems and experience a better quality of life during such critical times.

## Materials & Methods

### Design

This study employed a cross-sectional descriptive survey design to analyze the associations of sensitivity to COVID-19 infection and COVID-19 pandemic response indicators with psychosocial problems, as perceived by Korean nursing and non-nursing students. To this end, we set the following objectives: (1) comparison of the sensitivity levels, (2) comparison of the differences in sensitivity according to their general characteristics, (3) determination of the correlations between sensitivity, and (4) determination of the factors associated with psychosocial problems in nursing and non-nursing students.

### Participants

We determined the sample size required for intergroup comparison using G*Power software version 3.1.3 (University of Düsseldorf, Düsseldorf, Germany), applying the values of α = .05, power (1−β) = .80, and the effect size (*f*^2^) = 0.4 to the independent *t*-test analysis. The calculations yielded a sample size of 128 participants per group (nursing and non-nursing students). A total of 147 individuals per group were recruited in consideration of a 15% withdrawal rate. Eight undergraduate nursing students were excluded who provided consent but failed to submit the survey or gave incomplete responses. Hence, the final sample comprised 286 participants—139 nursing students and 147 non-nursing students.

### Data collection

Data were collected online between August 6 and October 30, 2020, from “S” university in North Chungcheong Province (Chungcheongbuk-do), Korea. Participants were recruited by convenience sampling using department bulletin boards and social media platforms (Facebook members who joined the “S” university page). In each document, we included a Google link to the questionnaire to allow interested students to participate in the survey. Separate documents and links were used to distinguish nursing from non-nursing students. The researcher and trained research assistant confirmed the eligibility of the participants. They were informed of the purpose of the study, and their consent was obtained. The invitation included a link that enabled the questionnaire to be activated, completed, and returned electronically. The questionnaire was online and self-reported, and the participants were given a small mobile-related gift to thank them for their participation. Response data were collected and stored using a questionnaire tool and subsequently exported for analysis. The questionnaire took approximately 10 min to complete. Unique visitors were determined based on their Google IDs. Insincere and biased responses were excluded.

### Questionnaire components

#### General characteristics

Participants’ general characteristics were categorized into sociodemographic characteristics and health-promoting behaviors. The former included age, gender, college year, and religion. The latter included smoking, drinking, chronic and respiratory diseases (presence or absence), vaccinations (that is, influenza, hepatitis A, hepatitis B, tetanus, and diphtheria) within 12 months (yes or no), hospital visits within three months (for cold, pre-existing disease treatment, vaccination, or COVID-19 test), perceived health status and self-isolation during the pandemic, and respiratory symptoms (yes or no).

#### Sensitivity to COVID-19 infection

To assess sensitivity to COVID-19 infection, we utilized items drawn from a comparative study conducted with community residents during the 2015 Middle East respiratory syndrome outbreak in South Korea (Lee et al., 2016). This instrument comprised the following five items concerning the reasons behind one’s sensitivity to COVID-19 infection: fear of COVID-19 infection, fear of death owing to aggravation of a pre-existing condition caused by COVID-19 infection, fear of COVID-19 infection in the family (that is, in children, seniors, and patients), fear of socioeconomic agitation owing to the rampant spread of COVID-19, and fear of COVID-19 in general. The items were rated on a five-point Likert-type scale ranging from 1 (*not at all*) to 5 (*extremely so*). The total score ranged from 5 to 25, with higher scores indicating higher sensitivity to COVID-19 infection. The Cronbach’s alpha for the overall reliability of the items for sensitivity to COVID-19 infection was .69.

#### COVID-19 pandemic response indicators

The COVID-19 pandemic response indicators were perceived stress, attitude toward preventive behaviors, compliance with handwashing, and trust in policy during the pandemic. The instrument was prepared based on a study conducted with community residents during the 2015 Middle East respiratory syndrome outbreak in South Korea (Lee et al., 2016).

We used one item to assess perceived stress on a four-point scale ranging from 1 (*not at all*) to 4 (*extremely so*), with a higher score indicating a higher level of perceived stress.

Attitude toward preventive behaviors was assessed using a seven-item instrument rated on a five-point scale, ranging from 1 (extremely non-compliant) to 5 (extremely compliant). This assessment was based on the guidelines of the Korea Disease Control and Prevention Agency (formerly known as the Korea Centers for Disease Control and Prevention). The total score ranged from 7 to 35, with higher scores indicating a more positive attitude toward preventive behaviors and compliance with prevention guidelines (Korea Disease Control and Prevention Agency, 2019).

Handwashing compliance was assessed using a four-item instrument rated on a four-point scale ranging from 1 (*not at all*) to 4 (*at all times*). The total score ranged from 4 to 16, with higher scores indicating better compliance.

Trust in policy was also assessed using a five-item instrument rated on a five-point scale, ranging from 1 (*not at all*) to 5 (*extremely so*). The total score ranged from 5 to 25, with higher scores indicating higher trust in the policy.

The Cronbach’s alpha for the overall reliability of the items for the COVID-19 pandemic response indicators was .65. The reliability value for attitude toward preventive behaviors, compliance with handwashing, and trust in policy was .50, .58, and .69, respectively.

#### Psychosocial problems

The Psychosocial Well-Being Index is a 45-item self-rating instrument, originally developed to assess psychosocial problems in adult workers and validated using the General Health Questionnaire-60 (Goldberg, 1978; Jang, 1993). In this study, we used the Psychosocial Well-Being Index Short Form, an 18-item questionnaire revised over two trials by Chang (2000). Items are rated on a four-point Likert-type scale ranging from 0 (*strongly agree*) to 3 (*strongly disagree*). Items 2, 3, 4, 7, 13, 15, and 16, which are negatively worded, are reverse-scored. Psychosocial health status is determined by the total score of the 18 items, which ranges from 0 to 54. The higher the total score, the lower the psychosocial health status. In Chang’s (2000) study, the Cronbach’s alpha of this questionnaire was .90. In this study, it was .92.

### Data analysis

Data analysis was performed using SPSS for Windows, version 25.0 (IBM Corp., Armonk, NY, USA). A two-tailed test was performed with a significance level (*α*) of .05. Intergroup differences in the levels of sensitivity to COVID-19 infection, COVID-19 pandemic response indicators, and psychosocial problems were determined using *t*-tests. Participants’ general characteristics (sociodemographic characteristics and health-promoting behaviors) were presented as frequency and percentage. Differences according to the general characteristics of each group were verified using *t*-tests, ANOVAs, and Scheffé’s post hoc tests. We used Pearson’s correlation analysis to determine the correlations between sensitivity to COVID-19 infection, COVID-19 pandemic response indicators, and psychosocial problems in nursing and non-nursing students and conducted multiple regression analyses to determine the factors associated with psychosocial problems in the two groups.

### Ethical considerations

For this study, we obtained approval from the Institutional Review Board of S University (IRB No. 2020-07-002-01). Before starting the study, all the participants completed written informed consent forms. The anonymity of the participants was guaranteed. This study was conducted following the principles outlined in the Declaration of Helsinki.

## Results

### Intergroup differences in sensitivity to COVID-19 infection, COVID-19 pandemic response indicators, and psychosocial problems

The non-nursing students were majoring in science and technology or liberal arts (law and humanities). The average age of the sample was 22.95 ± 2.02 years, and more women (80.8%), than men, participated in this study. A statistically significant intergroup difference (*p* = .008) was observed in sensitivity to COVID-19 infection (nursing students: 20.23 ± 3.09 points; non-nursing students: 19.16 ± 3.64 points). Among the COVID-19 pandemic response indicators, perceived stress showed no statistically significant intergroup difference (*p* = .264; nursing students: 4.06 ± 0.84, non-nursing students: 4.18 ± 0.85). Regarding attitudes toward preventive behaviors, the following items showed significant intergroup differences: “not touching the eyes, nose, and mouth with unwashed hands” (nursing students: 4.25 ± 0.87 *vs.* non-nursing students: 3.78 ± 1.01), “covering with a tissue or handkerchief when sneezing or coughing” (4.42 ± 0.74 *vs.* 4.14 ± 0.91), “wearing a mask when going out” (4.88 ± 0.04 *vs.* 4.74 ± 0.53), and “avoiding crowded places” (4.51 ± 0.87 *vs.* 3.82 ± 0.92). Regarding compliance with handwashing, no significant intergroup differences were observed (*p* = .945; nursing students: 15.34 ± 0.93, non-nursing students: 15.35 ± 1.12). Regarding trust in policy, the following items showed significant intergroup differences: “governmental COVID-19 prevention guidelines and public relations” (nursing students: 4.09 ± 0.85 *vs.*. non-nursing students: 3.84 ± 0.89), “ability of health providers to respond to COVID-19 infection” (3.95 ± 0.98 *vs.* 4.14 ± 0.96), and “governmental response to the COVID-19 pandemic” (3.71 ± 0.96 *vs.* 3.56 ± 1.12) (Table 1).

**Table 1:** Differences in sensitivity to COVID-19 infection, COVID-19 pandemic response indicators, and psychosocial problems according to the student department (*N* = 286).

Variables Categories	Nursing (*n* = 139) Mean ± SD	Non-nursing (*n* = 147) Mean ± SD	t (p)
Sensitivity to COVID-19 infection	20.23 ± 3.09	19.16 ± 3.64	2.680 (.008)*
Fear of COVID-19 infection	4.25 ± 0.86	3.86 ± 0.92	3.705 (.000)**
Fear of death owing to aggravation, caused by COVID-19 infection, of a pre-existing disease	3.73 ± 1.09	3.00 ± 1.36	5.030 (.000)**
Fear of COVID-19 infection in the family (i.e., in children, adults, and patients)	3.71 ± 1.28	4.31 ± 0.95	−4.511 (.000)**
Fear of socioeconomic agitation owing to the rampant spread of COVID-19	4.35 ± 0.83	3.99 ± 0.87	3.619 (.000)**
Fear of COVID-19 in general	4.19 ± 0.74	4.00 ± 0.81	2.013 (.045)*
COVID-19 pandemic response indicator: perceived stress	4.06 ± 0.84	4.18 ± 0.85	−1.119 (.264)
COVID-19 pandemic response indicator: attitude toward preventive behaviors	30.57 ± 2.87	30.07 ± 2.83	1.463 (.145)
Frequent handwashing with soap or hand sanitizer	4.46 ± 0.71	4.29 ± 0.80	−1.875 (.062)
Not touching the eyes, nose, and mouth with unwashed hands	4.25 ± 0.87	3.78 ± 1.01	4.226 (.000)**
Covering with a tissue or handkerchief when sneezing or coughing	4.42 ± 0.74	4.14 ± 0.91	−2.914 (.004)*
Avoiding contact with people with fever or respiratory symptoms			
Wearing a mask when going out	4.51 ± 0.83	4.50 ± 0.69	.157 (.875)
Avoiding crowded places			
Refraining from visiting hospitals and medical facilities	4.88 ± 0.04 4.51 ± 0.87 4.22 ± 0.79	4.74 ± 0.53 3.82 ± 0.92 4.13 ± 0.85	−2.461 (.014)* 6.485 (.000)** −.978 (.329)
COVID-19 pandemic response indicator: compliance with handwashing	15.34 ± 0.93	15.35 ± 1.12	−.069 (.945)
How often do you wash your hands before eating?	3.67 ± 0.50	3.68 ± 0.52	−.185 (.854)
How often do you wash your hands after using the bathroom?	3.97 ± 0.17	3.96 ± 0.23	.503 (.616)
How often do you wash your hands after coming back home?			
How often do you use soap or hand sanitizer?	3.89 ± 0.31 3.81 ± 0.44	3.90 ± 0.35 3.82 ± 0.42	−.151 (.880) −.066 (.947)
COVID-19 pandemic response indicator: trust in policy	19.58 ± 3.37	19.42 ± 3.95	.371 (.712)
Governmental COVID-19 prevention guidelines and public relations	4.09 ± 0.85	3.84 ± 0.89	2.354 (.019)*
Communication about the COVID-19 outbreak through the mass media	4.04 ± 0.93	3.95 ± 0.99	.798 (.426)
Ability of health providers to respond to COVID-19 infection	3.95 ± 0.98	4.14 ± 0.96	−2.404 (.017)*
Measures to prevent the spread of COVID-19 (quarantine, etc.)	3.88 ± 0.90	3.93 ± 1.04	−1.798 (.073)
Governmental response to the COVID-19 pandemic	3.71 ± 0.96	3.56 ± 1.12	2.478 (.014)*
Psychosocial problems	18.20 ± 10.34	18.62 ± 9.81	−.350 (.727)

**Notes.**

COVID-19: coronavirus disease 2019.

### Differences in psychosocial problems according to general characteristics by group

Table 2 outlines the differences in psychosocial problems according to the participants’ general characteristics. Women outnumbered men in both nursing (85.6% *vs.* 14.4%, respectively) and non-nursing students (76.2% *vs.* 23.8%, respectively). The prevalence of chronic diseases was 0.7% among nursing students and 7.5% among non-nursing students. The percentage of nursing students “vaccinated within 12 months” was twice of non-nursing students (73.4% *vs.* 30.6%, respectively). The number of hospital visits within three months was slightly higher among nursing than non-nursing students (52.5% *vs.* 48.3%, respectively).

**Table 2:** Differences in psychosocial problems according to nursing and non-nursing students’ general characteristics.

Variables	Categories	Nursing (*n* = 139)			Non-nursing (*n* = 147)		
		*n*(%)	Mean ± SD	t/F (p)	*n*(%)	Mean ± SD	t/F (p)
Gender	Men Women	20 (14.4) 119 (85.6)	17.70 ± 11.08 18.29 ± 10.25	.056 (.813)	35 (23.8) 112 (76.2)	15.85 ± 11.46 19.49 ± 9.12	3.726 (.056)
Grade	1 2 3 4	14 (10.1) 9 (6.5) 82 (59.0) 34 (24.5)	15.21 ± 9.05 23.77 ± 8.45 17.37 ± 10.87 19.97 ± 9.48	1.797 (.151)	15 (10.2) 40 (27.2) 30 (20.4) 62 (42.2)	21.26 ± 9.84 15.82 ± 9.58 20.93 ± 7.98 18.67 ± 10.46	2.045 (.110)
Religion	No religion Have religion	87 (47.0) 52 (53.0)	21.14 ± 9.71 14.92 ± 4.91	1.067 (.365)	98 (66.7) 49 (33.3)	21.88 ± 9.62 17.11 ± 10.17	2.626 (.053)
Smoking	No Yes	123 (88.5) 16 (11.5)	17.87 ± 10.64 20.75 ± 7.31	1.093 (.298)	113 (76.9) 34 (23.1)	18.25 ± 9.59 19.85 ± 10.56	.690 (.407)
Drinking (times/week)	0 1–2 3–4 ≥5	65 (46.8) 72 (51.8) 2 (1.4) 0 (0.0)	12.50 ± 10.51 17.58 ± 10.23 12.50 ± 10.60	.595 (.553)	67 (45.6) 66 (44.9) 12 (8.2) 2 (1.4)	13.41 ± 6.44 17.96 ± 9.22 19.89 ± 10.58 29.00 ±.0.00	2.413 (.069)
Chronic disease	No Yes	138 (99.3) 1 (0.7)	11.26 ± 10.36 18.00 ± 0.00	.488 (.486)	136 (92.5) 11 (7.5)	18.47 ± 10.14 20.45 ± 3.64	.411 (.522)
Respiratory disease	No Yes	129 (92.8) 10 (7.2)	18.14 ± 10.32 19.00 ± 11.11	.063 (.803)	131 (89.1) 16 (10.9)	18.55 ± 10.08 19.18 ± 7.48	.058 (.809)
Vaccination within 12 months	No Yes	37 (26.6) 102 (73.4)	20.00 ± 9.01 17.55 ± 10.74	1.519 (.220)	102 (69.4) 45(30.6)	19.47 ± 9.88 16.71 ± 9.48	2.495 (.116)
Hospital visits within three months	No Yes	66 (47.5) 73 (52.5)	17.48 ± 10.90 18.86 ± 9.83	.614 (.435)	76 (51.7) 71 (48.3)	16.03 ± 9.98 21.37 ± 8.88	11.737 (.001)*
PHS during COVID-19	Poor^a^ Normal^b^ Good^c^	6 (4.3) 40 (28.8) 93 (66.9)	24.50 ± 8.01 22.90 ± 10.32 15.78 ± 9.67	8.643 (.000) * b>c	9 (6.1) 48 (32.7) 90 (61.2)	32.22 ± 9.48 23.35 ± 6.56 14.74 ± 9.00	29.575 (.000) ** a>b>c
Self-isolation during COVID-19	No Yes	136 (97.8) 3 (2.2)	18.01 ± 10.35 27.00 ± 5.19	2.236 (.137)	134 (97.3) 4 (2.7)	18.78 ± 9.89 13.00 ± 3.46	1.355 (.246)
Respiratory symptoms	No Yes	130 (93.5) 9 (6.5)	18.19 ± 10.13 18.44 ± 13.76	.005 (.944)	129 (87.8) 18 (12.2)	17.75 ± 10.02 24.88 ± 4.78	8.804 (.004)*

**Notes.**

COVID-19: coronavirus disease 2019, PHS: perceived health status.

Scheffé’s post-hoc test results showed average differences in psychosocial problems between the perceived health status groups for nursing and non-nursing students. Nursing students showed a significantly higher prevalence of psychosocial problems with aggravated perceived health status during the COVID-19 pandemic (*F* = 8.643, *p* = .000). Regarding psychosocial problems, the average score for “Normal” was 22.90 points, while “Good” was lower at 15.78 points. Among non-nursing students, a statistically higher prevalence was associated with the following general characteristics: hospital visits within three months (*t* = 11.737, *p* = .001), lower perceived health status during the COVID-19 pandemic (*F* = 29.575, *p* < .001) and experienced respiratory symptoms (*t* = 8.804, *p* = .004). In psychosocial problems, the average score for “Normal” was 23.35 points, while the score for “Good” was lower at 14.74 points.

### Correlations between sensitivity to COVID-19 infection, COVID-19 pandemic response indicators, and psychosocial problems

Sensitivity to COVID-19 infection was positively correlated with attitude toward preventive behaviors (*r* = 0.332, *p* < .001). Perceived stress was positively correlated with trust in policy (*r* = 0.209, *p* < .001) and psychosocial problems (*r* = 0.158, *p* = .008). Compliance with handwashing was positively correlated with attitude toward preventive behaviors (*r* = 0.373, *p* < .001). Psychosocial problems were positively correlated with perceived stress (*r* = 0.158, *p* = .008) and trust in policy (*r* = 0.208, *p* < .001) and negatively correlated with attitude toward preventive behaviors (*r* =  − 0.116, *p* = .050). The findings are presented in Table 3.

**Table 3:** Correlations between sensitivity to COVID-19 infection, COVID-19 pandemic response indicators, and psychosocial problems.

Variables	Sensitivity to COVID-19 infection	COVID-19 pandemic response indicators				Psychosocial problems
		Perceived stress	Attitude toward preventive behaviors	Compliance with handwashing	Trust in policy	
	r(p)					
Sensitivity to COVID-19 infection	1					
Perceived stress	.077 (.197)	1				
Attitude toward preventive behaviors	.332 (.000)*	−.004 (.941)	1			
Compliance with handwashing	.110 (.064)	.111 (.060)	.373 (.000)*	1		
Trust in policy	082 (.165)	.209 (.000)*	.011 (.857)	.080 (.179)	1	
Psychosocial problems	.059 (.317)	.158 (.008)*	−.116 (.050)*	−.031 (.603)	.208 (.000)*	1

**Notes.**

COVID-19: coronavirus disease 2019.

### Factors associated with psychosocial problems in nursing and non-nursing students

Table 4 shows the multiple regression analysis results for the factors associated with psychosocial problems in nursing and non-nursing students. Independent variables included those verified to be significant in each group and the control variables. The input variables were gender, religion, drinking, respiratory symptoms (presence or absence), hospital visits within three months, and perceived health status during the COVID-19 pandemic.

**Table 4:** Results of the multiple regression analysis for the correlates of psychosocial problems by student department.

Variables	Nursing B	*p*	Non- medical B	*p*
COVID-19 pandemic response indicator: perceived stress	1.665	.112	1.263	.045*
COVID-19 pandemic response indicator: attitude toward preventive behaviors	−.060	.837	−.217	3.42
COVID-19 pandemic response indicator: trust in policy	.148	.568	.892	.000*
Gender (ref. men)	1.085	.662	1.494	.298
Religion (ref. no)	0.916	.183	−1.424	.004*
Drinking (ref. no)	.847	.629	−1.356	.122
Hospital visits within three months	.105	.952	1.932	.125
Perceived health status during the COVID-19 pandemic	−5.831	.000*	−8.513	.000*
Respiratory symptoms	−1.805	.618	.546	.798
R^2^	.142		.479	
Adjusted R^2^	.110		.461	
F (p)	4.398 (.001)*		25.965 (.000)*	

**Notes.**

COVID-19: coronavirus disease 2019.

A good model fit was obtained for the regression models in both nursing (*F* = 4.398, *p* = .001) and non-nursing students (*F* = 25.965, *p* < .001). The tolerance ranged from .76 to .89 for the nursing student model and from .90 to .99 for the non-nursing student model. Moreover, since the variance inflation factors for the variables of the nursing and non-nursing student models were assessed to be less than 10 (1.12–1.32 and 1.01–1.11, respectively), no multicollinearity was detected. The regression coefficients showed the changes in psychosocial problems for each variable while all other predictors remained constant. The *p*-values showed the significance of the corresponding regression coefficient. Traditionally, predictors with *p*-values equal to or less than 0.05 are considered significant in the regression model. A 95% confidence interval was considered in the present study.

Consequently, among the COVID-19 pandemic response indicators, stress was a significant variable for non-nursing students (β = 1.263, *p* = .045), as was the case for trust in policy (β = .892, *p* <  .001) and religion (β = −1.424, *p* = .004). Additionally, perceived stress was verified as a significant variable for both nursing (β =  − .32, *p* < .001) and non-nursing students (β =  − .49, *p* < .001).

## Discussion

This study examined the factors associated with Korean nursing and non-nursing students’ psychosocial problems during the COVID-19 pandemic. The results revealed that non-nursing students had more intense psychosocial problems than nursing students. These results are consistent with comparative studies on psychological problems between nursing and non-nursing students prior to the COVID-19 pandemic (Stewart, Greene & Coke, 2018; Zeng et al., 2019). Given the lack of studies on this topic during the COVID-19 pandemic, we have discussed the present results based on a more general comparison—between medical and non-medical students. In this context, the findings are consistent with those of Xie et al. (2020), which reported that non-medical students experience more severe social and psychological problems than medical students. Conversely, our results are inconsistent with those of Ye et al. (2020), which demonstrated that medical college students experience more severe psychosocial problems than non-medical students. Moreover, Xie et al. (2020) reported that medical students have more educational opportunities to gain specialized medical knowledge than non-medical college students, which reduces infection concerns. The results of this study also reflected that nursing students adhere to all preventive regulations compared to non-nursing students, excluding the areas of “handwashing,” “avoiding contact with symptomatic individuals,” and “refraining from visiting medical facilities.” These findings indicate that Korean nursing students comply with preventive measures based on their professional medical knowledge. However, this does not alleviate psychosocial problems such as concerns and anxieties about infection.

The psychosocial problems experienced by Korean non-nursing students were explained significantly by the proposed model, with an explanatory power of 46.1%. Specifically, COVID-19 pandemic-related stress, trust in policy, religion, and perceived health status during the COVID-19 pandemic were significantly associated with non-nursing students’ psychosocial problems. A study showed that people get stressed by various formal and informal information about COVID-19, pandemic-related changes in daily life, and uncertainties about the future. Consequently, stress can negatively affect their psychosocial health (Bao et al., 2020). In our study, a higher level of trust in policy for non-nursing students was associated with severe psychosocial problems. This contradicts Kecojevic et al.’s (2020) study, which showed that trust in public media could help alleviate psychosocial problems.

Awareness of the dangers of an epidemic or pandemic can lead to self-protective behaviors. This risk awareness helps prevent the spread of infectious diseases. However, excessive and fear-driven preventive behaviors can also cause psychosocial problems, such as overreaction and discrimination (Yang & Cho, 2017). Xie et al. (2020) reported that non-medical students experienced more severe psychosocial problems than medical students and suggested that the differences in psychosocial problems between them would increase over time. Xie et al. (2020) also mentioned that the continuation of the pandemic would result in psychosocial problem changes for these groups.

Kecojevic et al.’s (2020) study was based on survey data collected in April 2020. They stated that as time passed and coerced constraints on autonomy gradually grew (that is, through the enforcement of governmental policies to prevent the spread of infection), those who trusted the policies could eventually experience an increase in their risk perceptions toward COVID-19. This finding is contrary to that of a study conducted at the beginning of the pandemic (Ye et al., 2020). Moreover, the latter study showed that those who trusted the policies might have experienced psychosocial problems owing to the overreactions exacerbated by continued extreme self-control and excessive preventive behaviors (Ye et al., 2020). In nursing and non-nursing students, those with lower perceived health status had more serious psychosocial problems than their counterparts. This is consistent with the results of a study by Ruiz-Frutos et al. (2021). The study showed that among workers engaged in non-medical sectors during the COVID-19 pandemic, those with poor self-rated health status experienced more mental distress than those with good self-rated health status.

In this context, it seems important to use effective strategies to alleviate psychosocial problems, including anxiety (Nurunnabi et al., 2020; Bourion-Bédès et al., 2021). Studies have shown that religious coping helps increase resilience and self-efficacy. This, in turn, helps overcome life crises (Grover, Davuluri & Chakrabarti, 2014). Additionally, a study examining the factors associated with the quality of life of Malaysian university students during the COVID-19 pandemic showed that religious coping was highly correlated with an increase in the quality of life (Abdullah et al., 2020). Our evidence finds symmetry in these two studies, as religious students had fewer psychosocial problems than non-religious students. Religious individuals may be able to deal with psychosocial problems more positively through religious coping.

Nonetheless, the model that we proposed for the psychosocial problems of nursing students showed low explanatory power (11%). A significant part of nursing students’ psychosocial problems may be explained by variables we did not explore. Ulenaers et al. (2021) reported that the lack of professional practice due to the pandemic increased stress in nursing students. Fu et al. (2021) further reported that the stress and anxiety levels were high in college students engaged in practicum. It has also been reported that college students are experiencing psychological distress owing to experiencing “e-learning crack-up” and “fear of academic year loss” through online lectures (Hasan & Bao, 2020). Therefore, a follow-up study should further examine the correlates of psychosocial problems in nursing students. Preferably, this includes variables reflecting the nursing department’s characteristics, in which practical training is essential.

A stable learning environment is an important mitigating factor for the psychological problems of nursing students (Carolan et al., 2020). The imbalance between uncertainty and students’ ambition can be mitigated through regular communication with professors (Ulenaers et al., 2021). In addition, a steadily progressing curriculum per the schedule based on a long-term learning goal than a continuously changing short-term learning plan can help alleviate psychological problems by reducing uncertainty amongst students (Fitzgerald & Konrad, 2021; Haslam, 2021). Faculty members willing to participate in recognizing and referring at-risk students to additional resources and the structuring academic environment are also important mitigating factors for the psychological problems among non-nursing students (Kalkbrenner, Jolley & Hays, 2021). Additionally, internet-based interventions are effective for lowering distress among college students (Nguyen-Feng, Greer & Frazier, 2017). Therefore, a follow-up study should carefully investigate coping strategies and learning environments that work as mitigating factors.

### Limitations

This study has certain limitations. First, owing to the study’s cross-sectional design and its restriction to one region of South Korea, the results have low generalizability. Second, although we analyzed several variables, our proposed model only addressed 11% of the psychosocial problems faced by nursing students.

### Recommendations

For generalization, it is suggested that repeated studies are conducted based on various regions. Furthermore, it is necessary to investigate other potential correlates in a follow-up study, preferably by considering the role of other variables as identified in the previous studies. Based on this and the follow-up studies, there should be continuous efforts to provide college students with planned learning and supportive environments.

### Strengths

This study bridges a critical gap in research on Korean university students’ psychosocial problems during the COVID-19 pandemic. Specifically, it addresses the lack of research on the psychosocial problems of nursing and non-nursing college students. Additionally, our results highlight the importance of focusing on college students’ psychological health in coping with the COVID-19 pandemic and identifying high-risk groups among college students. This knowledge allows stakeholders to make well-informed decisions to develop intervention programs targeting these groups, which are consistent with their needs and, therefore, more effective.

## Conclusions

Perceived stress, trust in policy, religion, and perceived health status were significant correlates of psychosocial problems in non-nursing students. With nursing students, only perceived health status was a significant correlate. Our results highlight the necessity of identifying and managing college students at high risk of psychosocial problems during the COVID-19 pandemic. Future follow-up research is warranted to investigate the effects of potential influencing factors not considered in this study on psychosocial problems in nursing students. In addition, a replication study should be conducted with various subjects for generalization. Future studies should also explore efficient management strategies for psychosocial problems in university students during the COVID-19 pandemic, using the evidence presented in this paper as the primary data.

## Supplemental Information

10.7717/peerj.12541/supp-1 Supplemental Information 1 Raw data

## Additional Information and Declarations

### Data Availability

The following information was supplied regarding data availability: The raw measurements are available in the Supplementary File.
